# Histopathological Correlation in Suspected Celiac Disease: Linking Clinical, Serological, and Endoscopic Findings

**DOI:** 10.12688/f1000research.173342.1

**Published:** 2025-12-08

**Authors:** Genan AlMajed, Reem Alateeq, Sarah Bubshait, Manar Barnawi, Razan Almadani, Awadia Awadalla, Ahmed Alsayyah, Liqa Almulla

**Affiliations:** 1 Imam Abdulrahman Bin Faisal University College of Medicine, Dammam, Eastern Province, Saudi Arabia

**Keywords:** Celiac disease, duodenal biopsy, histopathological correlation, serological markers, Marsh classification

## Abstract

Celiac disease (CD) is an immune-mediated enteropathy triggered by gluten in genetically susceptible individuals, characterized by villous atrophy, crypt hyperplasia, and intraepithelial lymphocytosis. Despite advances in serological testing, duodenal biopsy remains the diagnostic gold standard, especially in atypical or patchy disease. This retrospective study at King Fahd University Hospital (KFUH), Saudi Arabia, evaluated the diagnostic contribution of biopsies from the duodenal bulb (D1) and distal duodenum (D2) in 224 patients assessed for suspected CD between August 2023 and August 2024. Clinical, serological [IgA anti-tissue transglutaminase (anti-TG2), anti-endomysial antibodies (EMA), deamidated gliadin peptide], and histopathological data were analyzed. Sixty-five patients (29%) were diagnosed with CD. Villous atrophy, mucosal flattening, and increased intraepithelial lymphocytes were strongly associated with CD (p ≤ 0.001), and positive anti-TG2 and EMA correlated with Marsh grade. Combined D1 and D2 sampling improved diagnostic yield, underscoring the value of integrating histology and serology in evaluating classical and non-classical CD presentations.

## Introduction

CD is an immune-mediated enteropathy triggered by gluten in genetically predisposed individuals, causing villous atrophy. It may present with gastrointestinal (GI) symptoms, extra-intestinal signs, or be asymptomatic, making diagnosis challenging and increasing complication risks. ^1, 2^ These variations highlight the need for comprehensive diagnostic evaluation, including histopathological, serological, and endoscopic testing. ^2^

CD is common affecting about 1% of the global population. ^3^ However, the majority of cases are underdiagnosed due to the non-specific and variable clinical presentations. ^4, 5^ Advances in diagnostics, greater awareness, and shifts in gluten exposure and infant feeding have improved understanding of CD. ^6, 7^ In Saudi Arabia, prevalence rates reach 3.2% in Al-Qaseem, 2.1% in Aseer, and 1.8% in Madinah. ^8, 9^

Since the early 1980s, serum autoantibody testing has been a key diagnostic tool for CD and remains valuable, particularly for screening symptomatic patients. ^10^ However, due to variability in serological and endoscopic sensitivity and specificity, duodenal biopsies remain the diagnostic gold standard. ^11^

Since the 1990s, duodenal biopsies from D2 have become standard with advances in endoscopy. ^12^ Emerging evidence shows that D1 biopsies may be equally or even more valuable, as the duodenal bulb often exhibits the earliest and most severe mucosal damage due to its high exposure to gluten, acid, and pepsin. ^13^ Some cases report isolated D1 involvement, highlighting its diagnostic importance. ^9^

The recognition that CD may affect the small intestine unevenly has led to recommendations for multiple biopsy specimens during esophagogastroduodenoscopy (EGD) when CD is suspected. ^12^ The optimal biopsy number and sites remain debated, but guidelines recommend sampling D2 and D1 to improve yield. ^8^

Serological markers like IgA anti-TTG and EMA are crucial for screening and early diagnosis of CD. Anti-TTG is highly sensitive and specific, while EMA offers 100% specificity, making it a valuable confirmatory marker. ^14^ In a Saudi cohort, high anti-TTG titers (≥10 x upper limit of normal (ULN)) combined with positive EMA enabled non-biopsy diagnosis in 57.3% of suspected CD cases. ^9^ However, omitting biopsy should be approached cautiously due to the variability in serological test results across populations. ^15^

Histopathological evaluation of duodenal biopsies remains the gold standard for diagnosing CD, with the Modified Marsh classification ranging from Type 0 (normal mucosa) to Type 3 (complete villous atrophy). Correlating these changes with serological markers is crucial for an accurate diagnosis. ^8^ Variations in Marsh grading between D1 and D2 biopsies suggest patchy disease distribution, highlighting the need for multiple biopsy sites. ^12^

Despite diagnostic advances, the role of duodenal biopsies in CD diagnosis remains an area of active investigation. ^9^ This study focuses on evaluating the diagnostic practices and outcomes related to duodenal biopsies for suspected CD cases at KFUH, assessing their correlation with clinical, serological, and endoscopic findings in both typical and atypical presentations.

## Objectives of the study

• **Primary Objective:** Evaluate the diagnostic utility of duodenal biopsies in adult and pediatric populations with suspected CD within KFUH.
• **Secondary Objectives:**
  ▪ Assess the correlation between endoscopic and histopathological findings from duodenal biopsies, and serological findings with the clinical symptoms presented by these patients.
  ▪ Determine the prevalence of duodenal biopsy involvement in patients diagnosed with CD.
  ▪ Analyze the variation in biopsy outcomes among patients exhibiting different symptom profiles, including GI, dermatological, neurological and asymptomatic cases.
  ▪ Investigate the influence of demographic factors, such as age and gender and examine their impact on the prevalence of CD and biopsy results within this patient population.

## Materials and methods

***Study design and Setting:*** This study is a retrospective cohort study conducted at KFUH, AlKhobar, Saudi Arabia, utilizing data from adult and pediatric patients with suspected CD who underwent duodenal biopsy.

***Subject: Participants, recruitment and sampling procedure:*** The population of interest for the study is all male and female patients of all ages with suspension of CD who underwent a duodenal biopsy within the GI clinic of KFUH between August 2023 and August 2024.

***Sample size*:** To find the bare minimum of representatives needed to accurately reflect the population in the sample size, sample size calculations were performed. The Raosoft sample size calculator was used to calculate the sample size. The estimated sample size required to study the prevalence of biopsy-proven CD in Saudi Arabia, with a 95% confidence interval, indicator percentage of 0.50, and a 5% margin of error, is approximately 385 patients.

***Inclusion and exclusion criteria:*** This study included patients of all ages who presented with signs and symptoms suggestive of CD and underwent a duodenal biopsy within the GI clinic of KFUH. Patients with incomplete biopsy data, patients with other pre-existing GI conditions were not included in this study.

**Method for data collection:** Hospital electronic medical records (EMR) and patient charts, histopathological reports from the pathology department of the hospital and laboratory reports from the hospital’s clinical laboratory.

**Instrument:** Data Collection Template (DCT), which includes structured fields for recording and scoring clinical symptoms, endoscopic, and histopathological findings according to the Marsh classification, and interpretations of serological markers. This template ensured systematic data extraction from hospital records, facilitating consistent and comprehensive documentation of all relevant information for each patient while ensuring confidentiality and patient’s privacy.

**Statistical analysis plan:** The collected data were extracted, reviewed, and coded using Microsoft Excel before being imported into IBM SPSS Statistics, version 29 (IBM Corp., Armonk, NY, USA) for analysis. Descriptive statistics were used to summarize the data: numerical variables were presented as mean ± standard deviation (SD) or median with interquartile range (IQR), depending on their distribution, while categorical variables were reported as frequencies and percentages. Diagnostic accuracy of endoscopic findings was assessed using 2×2 tables, calculating indices such as sensitivity and specificity, with histopathology considered the gold standard. Group comparisons were performed using the Student’s t-test and one-way ANOVA, depending on data distribution. Normality was assessed using the Kolmogorov–Smirnov test. Associations between categorical variables were examined using the Chi-squared test or Fisher’s exact test, as appropriate. A *p-value* <0.05 was considered statistically significant.

### Ethical considerations

This retrospective study was reviewed and approved by the Institutional Review Board of Imam Abdulrahman bin Faisal University, Dammam, Saudi Arabia (IRB Number: IRB-UGS-2024-01-693; approval date: October 8, 2024). The IRB granted a waiver of written informed consent because the study involved retrospective analysis of anonymized patient data, and obtaining consent was not feasible. All procedures adhered to institutional ethical standards and the Declaration of Helsinki (2013 revision). Patient confidentiality and data privacy were strictly maintained.

## Results

This study included 224 adult and pediatric patients with suspected celiac disease (CD) from KFUH, AlKhobar, Saudi Arabia. Mean age was 40.9 ± 16.5 years (range: 6–87). Most were Saudi (89.7%) and female (56.7%). Only 2 (0.9%) reported a family history of CD. Malabsorption symptoms were present in 64.3%, and 29% had extra-intestinal manifestations. Comorbidities (e.g., type 1 diabetes, thyroid disorders, inflammatory bowel diseases (IBD), psoriasis) were reported in 16.1%, while 72.3% had none ( Table 1).

**Table 1.** Demographic and clinical characteristics of the participants (n = 224).

Variable	Categories	N (%)
Gender	Male	97 (43.3)
	Female	127 (56.7)
Nationality	Saudi	201 (89.7)
	Non-Saudi	22 (9.8)
	Missing *	1 (0.4)
Family history of CD	Yes	2 (0.9)
	No	35 (15.6)
	Missing *	187 (83.5)
Malabsorption symptoms ^†^	Yes	144 (64.3)
	No	54 (24.1)
	Missing *	26 (11.6)
Extra-intestinal manifestations ^‡^	Yes	65 (29)
	No	110 (49.1)
	Missing *	49 (21.9)
Other comorbidities	Yes	36 (16.1)
	No	162 (72.3)
	Missing *	26 (11.6)

N: Number, %: Percentage, CD: Celiac disease.

* Unavailable data.

^†^ Defined as diarrhea, weight loss, abdominal pain or bloating.

^‡^ Includes anemia, dermatitis herpetiformis, osteopenia or osteoporosis.

Most biopsies were from the duodenum (non-specific) (n = 204, 91.1%). No villous atrophy was seen in 186 participants (83%), while nonspecific atrophy was noted in 28 (12.5%). Mucosal flattening and scalloping appeared in 11 (4.9%) and 8 (3.6%), respectively. Increased intraepithelial lymphocyte (IEL) infiltration was absent in 143 (63.8%), with mild increase (25–29 IELs/100 enterocytes) in 30 (13.4%). Marsh changes were seen in 26 (11.6%), crypt hyperplasia in 16 (7.1%), villous atrophy in 36 (16.1%), and lymphocyte infiltration in 55 (24.6%) ( Table 2).

**Table 2.** Histological and clinical markers for celiac disease assessment.

Variable	Categories	N (%)
Site of biopsy *	Duodenum (non-specific)	204 (91.1)
	Both (D1 ^†^ and D2 ^†^)	20 (8.9)
Villous atrophy	Yes	31 (13.8)
	No	186 (83)
	Missing ^‡^	7 (3.1)
Villous atrophy	Not specific	28 (12.5)
	Specific in D1 ^†^ & not in D2 ^†^	1 (0.4)
	Specific in D2 ^†^ & not in D1 ^†^	2 (0.9)
	No villous atrophy	186 (83)
	Missing ^‡^	7 (3.1)
Mucosal flattening	Yes	11 (4.9)
	No	200 (89.3)
	Missing ^‡^	13 (5.8)
Visible scalloping of duodenal folds	Yes	8 (3.6)
	No	204 (91.1)
	Missing ^‡^	12 (5.4)
Mosaic pattern	Yes	0 (0)
	No	211 (94.2)
	Missing ^‡^	13 (5.8)
Intraepithelial lymphocytes (IEL) count/100 entrocytes *	Normal	9 (4)
	Borderline or mild increase	30 (13.4)
	Increased IELs (Suggestive of pathology)	2 (0.9)
	No evidence of increased IEL	143 (63.8)
	Missing ^†^	40 (17.9)
Intraepithelial lymphocytes (IEL)	Yes	41 (18.3)
	No	143 (63.8)
	Missing ^†^	40 (17.9)
Marsh classification	Yes	26 (11.6)
	Missing ^†^	198 (88.4)
Presence of crypt hyperplasia	Yes	16 (7.1)
	No	201 (89.7)
	Missing ^†^	7 (3.1)
Degrees of villous atrophy	Yes	36 (16.1)
	No	38 (17)
	Missing ^†^	150 (67)
Lymphocyte infiltration	Yes	55 (24.6)
	No	165 (73.7)
	Missing ^†^	4 (1.8)

N: Number, %: Percentage.

* Biopsy site recorded by endoscopy report. Normal: <25 IELs/100 Enterocytes, Borderline or Mild Increase: 25-29 IELs/100 enterocytes, Increased IELs (Suggestive of pathology): 30 IELs/100 enterocytes.

^†^ D1: Duodenal bulb, D2: Distal duodenum.

^‡^ Unavailable data.

IgA anti-TG2 was positive in 11 participants (4.9%), and IgA EMA in 6 (2.7%). Total IgA was normal in 35 (15.6%) and abnormal in 4 (1.8%). All tested for IgG EMA and anti-DGP were negative (12.1% and 0.4%, respectively), while 4 (1.8%) were positive for both IgG anti-TG2 and anti-DGP ( Table 3).

**Table 3.** Serological markers for celiac disease assessment.

Variable	Categories	N (%)
IgA Anti-TG2 *	Positive	11 (4.9)
	Borderline/Equivocal	1 (0.4)
	Negative	33 (14.7)
	Missing ^†^	179 (79.9)
IgA EMA ^‡^	Positive	6 (2.7)
	Negative	34 (15.2)
	Missing ^†^	184 (82.1)
Total IgA levels	Normal	35 (15.6)
	Abnormal	4 (1.8)
	Missing ^†^	185 (82.6)
DGP	Negative	1 (0.4)
	Missing ^†^	223 (99.6)
IgG EMA ^‡^	Negative	27 (12.1)
	Missing ^†^	197 (87.9)
Anti-DGP ^§^	Positive	4 (1.8)
	Borderline/Equivocal	1 (0.4)
	Negative	17 (7.6)
	Missing ^†^	202 (90.2)
IgG anti-TG2 *^||^*	Positive	4 (1.8)
	Borderline/Equivocal	1 (0.4)
	Negative	41 (18.3)
	Missing ^†^	178 (79.5)

N: Number, %: Percentage. TG2, Tissue transglutaminase; EMA, Endomysial antibodies; DGP, Deamidated gliadin peptide; Ig, Immunoglobulin.

* IgA TG2: Negative: <4U/mL, Borderline/Equivocal: 4-10 U/mL, Positive: >10 U/mL;

^†^ Unavailable data;

^‡^ IgA or IgG EMA: Positive: at any titer level;

^§^ Anti-DGP: Negative: <20 U/mL, Positive: >30U/mL;

^||^ IgG TG2: Negative: <6U/mL, Borderline/Equivocal: 6-9 U/mL, Positive: >9 U/mL.

**Figure 1.**
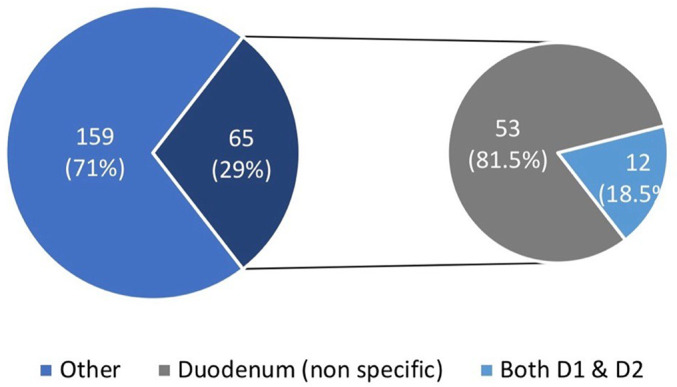
Prevalence of duodenal biopsy involvement in CD patients.

CD was diagnosed in 29% of participants (n = 65), while 71% (n = 159) had other conditions, including gastritis, and duodenitis. Among CD cases, 53 patients (81.5%) underwent non-specified duodenal biopsy, whereas in a distinct group of 12 patients (18.5%), the endoscopy reports specifically documented biopsies from both D1 and D2 segments ( Figure 1).

**Figure 2.**
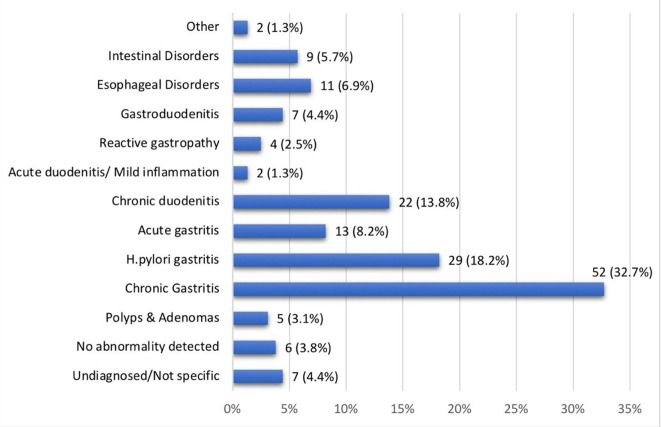
Final diagnoses other than Celiac Disease.

Chronic gastritis, including antral and hemorrhagic types, was the most common diagnosis in non-celiac patients (n = 52, 32.7%), followed by *Helicobacter pylori* gastritis (n = 29, 18.2%), chronic duodenitis (n = 22, 13.8%), acute gastritis (n = 13, 8.2%), and esophageal disorders like GERD, eosinophilic esophagitis, and acute esophagitis (n = 11, 6.9%) ( Figure 2).

CD diagnosis was significantly associated with endoscopic and histological features including villous atrophy, non-specific villous atrophy, mucosal flattening, duodenal scalloping, and mild IEL increase ( *p = 0.001*). It was also linked to crypt hyperplasia, varying degrees of villous atrophy, and lymphocyte infiltration ( *p = 0.003, <0.001*, and *0.009*, respectively). Positive or borderline IgA anti-TG2 and IgG anti-TG2 levels were significantly associated with CD ( *p* < 0.001 and *p* = 0.003). Other factors showed no significant association with final diagnosis ( *p* > 0.05) ( Table 4).

**Table 4.** Factors associated with final diagnosis.

Variable	Categories	Final diagnosis		* p*
		Other	Celiac disease	
		N (%)		
Villous atrophy	Yes	15 (9.5)	**16 (27.1)**	**0.001** ^†^
	No	143 (90.5)	43 (72.9)	
Villous atrophy	Not specific	13 (8.2)	**15 (25.4)**	**0.001** ^‡^
	Specific in D1 ^§^ & not in D2 ^§^	0 (0)	1 (1.7)	
	Specific in D2 ^§^ & not in D1 ^§^	2 (1.3)	0 (0)	
	No villous atrophy	143 (90.5)	43 (72.9)	
Mucosal flattening	Yes	3 (1.9)	**8 (14.5)**	**0.001** ^‡^
	No	153 (98.1)	47 (85.5)	
Visible scalloping of duodenal folds	Yes	0 (0)	**8 (14.5)**	**< 0.001** ^‡^
	No	157 (100)	47 (85.5)	
Intraepithelial lymphocytes count/100 enterocytes *	Normal	8 (6.6)	1 (1.6)	**< 0.001** ^‡^
	Borderline or mild increase	8 (6.6)	**22 (34.9)**	
	Increased IELs	2 (1.7)	0 (0)	
	Lack of documentation of IEL	103 (85.1)	40 (63.5)	
Intraepithelial lymphocytes	Yes	18 (14.9)	**23 (36.5)**	**0.001** ^†^
	No	**103 (85.1)**	40 (63.5)	
Presence of crypt hyperplasia	Yes	6 (3.9)	**10 (16.1)**	**0.003** ^‡^
	No	149 (96.1)	52 (83.9)	
Degrees of villous atrophy	Yes	14 (26.9)	**22 (100)**	**< 0.001** ^†^
	No	38 (73.1)	0 (0)	
Lymphocyte infiltration	Yes	32 (20.3)	**23 (37.1)**	**0.009** ^†^
	No	126 (79.7)	39 (62.9)	
IgA Anti-TG2 *	Positive	1 (3.4)	**10 (62.5)**	**< 0.001** ^**‡**^
	Borderline/Equivocal	0 (0)	**1 (6.3)**	
	Negative	28 (96.6)	5 (31.3)	
IgA EMA ^§^	Positive	2 (8.7)	4 (23.5)	0.373 ^‡^
	Negative	21 (91.3)	13 (76.5)	
Total IgA levels	Normal	24 (88.9)	11 (91.7)	1.000 ^‡^
	Abnormal	3 (11.1)	1 (8.3)	
Anti-DGP ^||^	Positive	1 (9.1)	3 (27.3)	0.311 ^‡^
	Borderline/Equivocal	0 (0)	1 (9.1)	
	Negative	10 (90.9)	7 (63.6)	
IgG Anti-TG2 **	Positive	0 (0)	**4 (25)**	**0.003** ^‡^
	Borderline/Equivocal	0 (0)	**1 (6.3)**	
	Negative	30 (100)	11 (68.8)	

N: Number, %: Percentage.

TG2, Tissue transglutaminase; EMA, Endomysial antibodies; DGP, Deamidated gliadin peptide; Ig, Immunoglobulin.

* Intraepithelial lymphocytes (Normal: < 25 IELs/100 enterocytes, Borderline or Mild Increase: 25-29 IELs/100 enterocytes, Increased IELs (Suggestive of pathology): ≥ 30 IELs/100 enterocytes), gA TG2: Negative: <4 U/mL, Borderline/Equivocal: 4-10 U/mL, Positive: >10 U/mL;

^†^ Chi-square test,

^‡^ Fisher’s exact test;

^§^ D1: duodenal bulb; D2: distal duodenum; IgA or IgG EMA: Positive: at any titer level;

^||^ Anti-DGP: Negative: <20 U/mL, Positive: >30 U/mL;

** IgG TG2: Negative: <6 U/mL, Borderline/Equivocal: 6-9 U/mL, Positive: >9 U/mL.

**Figure 3.**
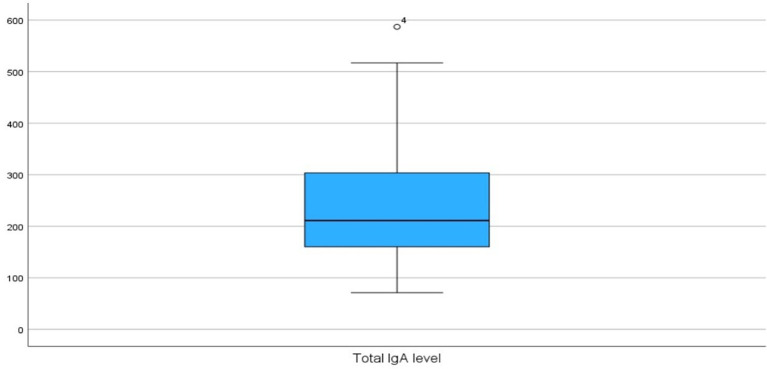
Total IgA level.

The mean of total IgA level was 240 ± 119 (Range: 71 – 587) with a median of 211 (IQR: 159 – 310) ( Figure 3).

No significant association was observed between clinical features such as malabsorptive symptoms, extra-intestinal manifestations, or comorbidities, and endoscopic or histological findings including villous atrophy, mucosal flattening, IEL count, crypt hyperplasia, or duodenal scalloping ( *p > 0.05*). In the overall study population, mucosal flattening was significantly more common in males ( *p = 0.012*), with no association found with age. Other features, including villous atrophy, IEL count, and crypt hyperplasia, showed no significant correlation with gender or age ( *p > 0.05*).

In the CD subgroup, mucosal flattening remained significantly more prevalent among males ( *87.5%, p = 0.003*), while other histological and endoscopic features showed no association with gender or age ( *p > 0.05*). Similarly, clinical findings like malabsorption and extra-intestinal symptoms were not significantly related to histological or endoscopic changes ( *p > 0.05*).

Significant associations were found between serological markers and histological features. Positive IgA anti-TG2 was associated with specific villous atrophy in D1, mucosal flattening, and a borderline or mild IEL increase ( *p = 0.001, 0.003*, and *0.001*, respectively). Positive anti-DGP was linked to non-specific villous atrophy, IEL increase, and crypt hyperplasia ( *p = 0.039, 0.007, and 0.004*). Positive IgA EMA also showed significant associations with non-specific villous atrophy, IEL increase, and crypt hyperplasia ( *p = 0.028, 0.020*, and *0.011*) ( Table 5).

**Table 5.** Correlation between serological markers, endoscopic and histological features.

Variable	IgA Anti-tissue trans-glutaminase			Anti-deamidated gliadin peptide		
	Positive	Negative	Borderline/Equivocal	Positive	Negative	Borderline/Equivocal
	N (%)					
**Villous atrophy:**						
No villous atrophy	1 (3.8)	** 25 (96.2)**	-	1 (7.1)	**13 (92.9)**	0 (0)
Not specific	**9 (56.3)**	7 (43.8)	-	**3 (37.5)**	4 (50)	**1 (12.5)**
Specific in D1 * & not in D2 *	**1 (100)**	0 (0)	-	-	-	-
Specific in D2 * & not in D1 *	0 (0)	**1 (100)**	-	-	-	-
***p***	**< 0.001**			**0.039**		
**Mucosal flattening:**						
Yes	**3 (60)**	1 (20)	**1 (20)**	1 (20)	3 (60)	1 (20)
No	5 (13.9)	**31 (86.1)**	0 (0)	2 (12.5)	14 (87.5)	0 (0)
***p***	**0.003**			0.228		
**Intraepithelial lymphocytes (IEL)** ^†^ **:**						
Lack of documentation of IEL	0 (0)	**21 (100)**	0 (0)	0 (0)	**10 (100)**	0 (0)
Normal	0 (0)	**3 (100)**	0 (0)	0 (0)	**3 (100)**	0 (0)
Borderline or mild increase	**10 (66.7)**	4 (26.7)	1 (6.7)	**4 (57.1)**	2 (28.6)	1 (14.3)
Increased IELs	-	-	-			
***p***	**< 0.001**			**0.007**		
**Presence of crypt hyperplasia:**						
Yes	4 (57.1)	3 (42.9)	0 (0)	**3 (75)**	1 (25)	0 (0)
No	6 (17.6)	27 (79.4)	1 (2.9)	0 (0)	**15 (93.8)**	**1 (6.3)**
***p***	0.096			**0.004**		

Variable:	IgA Anti-endomysial antibodies (EMA)		IgG Anti-tissue transglutaminase (anti-TG2)			Total IgA levels	
	Positive	Negative	Positive	Negative	Borderline/Equivocal	Normal	Abnormal
	N (%)						
**Villous atrophy:**							
No villous atrophy	1 (3.6)	27 (964)	0 (0)	**27 (100)**	-	22 (88)	3 (12)
Not specific	**4 (44.4)**	5 (55.6)	**4 (26.7)**	11 (73.3)	-	11 (91.7)	1 (8.3)
Specific in D1 * & not in D2 *	0 (0)	**1 (100)**	0 (0)	**1 (100)**	-	-	-
Specific in D2 * & not in D1 *	0 (0)	**1 (100)**	0 (0)	**1 (100)**	-	1 (100)	0 (0)
***p***	**0.028**		**0.023**		-	1.000	
**Mucosal flattening:**							
Yes	2 (33.3)	4 (66.7)	**2 (50)**	2 (50)	-	6 (100)	0 (0)
No	2 (6.3)	30 (93.8)	2 (5.4)	**35 (94.6)**	-	26 (86.7)	4 (13.3)
***p***	0.110		**0.041**		-	1.000	
**Intraepithelial lymphocytes (IEL)** ^†^ **:**							
Lack of documentation of IEL	1 (4.3)	**22 (95.7)**	0 (0)	22 (95.7)	1 (4.3)	18 (85.7)	3 (14.3)
Normal	0 (0)	**3 (100)**	0 (0)	3 (100)	0 (0)	3 (100)	0 (0)
Borderline or mild increase	**4 (44.4)**	5 (55.6)	3 (21.4)	11 (78.6)	0 (0)	11 (91.7)	1 (8.3)
***p***	**0.020**		0.117		-	1.000	
**Presence of crypt hyperplasia:**							
Yes	**3 (60)**	2 (40)	**3 (42.9)**	4 (57.1)	0 (0)	6 (100)	0 (0)
No	2 (6.1)	**31 (93.9)**	1 (2.8)	**34 (94.4)**	1 (2.8)	27 (90)	3 (10)
***p***	**0.011**		**0.010**			1.000	

N: Number, %: Percentage, p- values calculated using Fisher’s exact test;

* D1: Duodenal bulb; D2: Distal duodenum;

^†^ Intraepithelial lymphocytes (Normal: < 25 IELs/100 enterocytes, Borderline or Mild Increase: 25-29 IELs/100 enterocytes, Increased IELs (Suggestive of pathology): ≥ 30 IELs/100 enterocytes).

Among patients positive or equivocal for **IgA anti-TG2**, 91.7% (n = 11) showed borderline/mild IEL increase and met Marsh criteria. Crypt hyperplasia was found in 33.3% (n = 4), and all (n = 12) had villous atrophy with lymphocyte infiltration.

Among **EMA**-positive patients, 66.7% (n = 4) had borderline/mild IEL increase, 83.3% (n = 5) met Marsh criteria, 50% (n = 3) had crypt hyperplasia, and 83.3% (n = 5) showed villous atrophy with lymphocyte infiltration.

Of those with positive **total IgA**, 75% (n = 3) lacked IEL data. One participant (25%) met Marsh criteria and had lymphocyte infiltration. None had crypt hyperplasia or villous atrophy.

All **anti-DGP** positive patients (n = 5) had borderline/mild IEL increase, fulfilled Marsh criteria, showed villous atrophy with lymphocyte infiltration; 60% (n = 3) had crypt hyperplasia.

Among **IgG anti-TG2** positive/equivocal patients, 60% (n = 3) had borderline/mild IEL increase, 80% (n = 4) met Marsh criteria, and 80% (n = 4) showed villous atrophy with lymphocyte infiltration. Crypt hyperplasia was present in 60% (n = 3) ( Table 6).

**Table 6.** Histological findings of seropositive patients.

Variable	+ve for IgA anti-TG2 (n = 12)	+ve for EMA (n = 6)	+ve for total IgA levels (n = 4)	+ve for Anti-DGP (n = 5)	+ve for IgG anti-TG2 (n = 5)
	N (%)				
**Intraepithelial lymphocytes count:**					
Borderline or mild increase	11 (91.7)	4 (66.7)	1 (25)	5 (100)	3 (60)
Lake of documentation of IEL	0 (0)	1 (16.7)	3 (75)	0 (0)	1 (20)
Missing ^†^	1 (8.3)	1 (16.7)	0 (0)	0 (0)	1 (20)
**Marsh classification:**					
Yes	11 (91.7)	5 (83.3)	1 (25)	5 (100)	4 (80)
Missing ^†^	1 (8.3)	1 (16.7)	3 (75)		1 (20)
**Presence of crypt hyperplasia:**					
Yes	4 (33.3)	3 (50)	0 (0)	3 (60)	3 (60)
No	7 (58.3)	2 (33.3)	3 (75)	1 (20)	2 (40)
Missing ^†^	1 (8.3)	1 (16.7)	1 (25)	1 (20)	0 (0)
**Degrees of villous atrophy:**					
Yes	12 (100)	5 (83.3)	0 (0)	5 (100)	4 (80)
No	0 (0)	0 (0)	1 (25)	0 (0)	0 (0)
Missing ^†^	0 (0)	1 (16.7)	3 (75)	0 (0)	1 (20)
**Lymphocyte infiltration:**					
Yes	12 (100)	5 (83.3)	1 (25)	5 (100)	4 (80)
No	0 (0)	1 (16.7)	3 (75)	0 (0)	1 (20)

N: Number, %: Percentage.

* Intraepithelial lymphocytes (Normal: < 25 IELs/100 enterocytes, Borderline or Mild Increase: 25-29 IELs/100 enterocytes, Increased IELs (Suggestive of pathology): ≥ 30 IELs/100 enterocytes),

^†^ Unavailable data.

**Figure 4.**
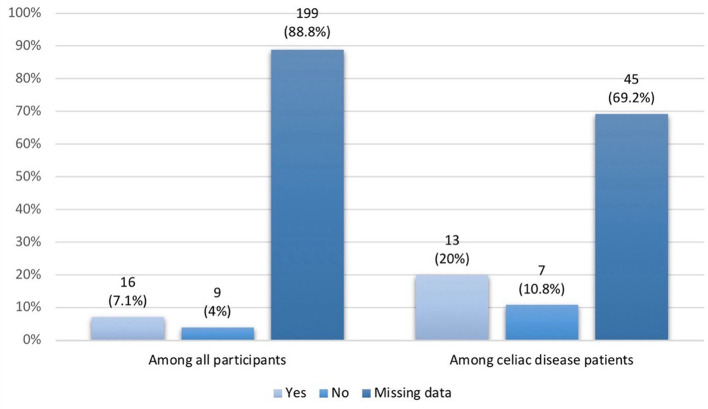
Response to gluten free diet.

The results indicated that the overall response rate to a gluten-free diet among all study participants (n = 224) was 7.1% (n = 16). However, a higher response rate was observed among patients with celiac disease (n = 65), reaching 20% (n = 13) ( Figure 4).

## Discussion

This study aimed to assess the diagnostic value of duodenal biopsies in suspected CD cases at KFUH. Most participants were Saudi (89.7%) and female (56.7%), reflecting global trends of higher CD prevalence in females. ^16^ In our study, 83% had no villous atrophy, 16.1% had definite atrophy, and 12.5% showed non-specific atrophy. Normal IEL counts were seen in 63.8%. This practice aligns with recommendations for multiple biopsy sites to improve diagnostic sensitivity, as CD lesions can be patchy and vary in distribution. ^17^ Additionally, guidelines suggest that taking four to six biopsy specimens from the descending duodenum is advisable. ^18^

In our cohort, 4.9% tested positive for IgA anti-TG2 antibodies, with 0.4% showing borderline levels. IgA anti-TG2 antibodies are key in CD diagnosis. Ben-Tov et al. (2025) showed that high-positive levels (≥10 × ULN) strongly correlate with EMA positivity, suggesting repeat anti-TG2 testing may replace EMA as confirmation. ^19^ The normal total IgA levels observed in 15.6% of participants are noteworthy. Monitoring IgA is essential, as deficiency, which is seen in 1–2% of CD cases, may yield false-negative IgA-based serology. ^20^

In our cohort, CD was diagnosed in 29% (65 participants), while 71% (159) had other GI conditions, underscoring the broader diagnostic utility of duodenal biopsies. Similarly, Demir et al. reported CD in 3.2% of patients with iron deficiency anemia, demonstrating the value of routine biopsies in detecting diverse pathologies. ^21^ Of the 65 CD patients, 81.5% had duodenal biopsies with unspecified locations, whereas 18.5% had documented sampling from both D1 and D2 segments in the endoscopy reports. This is clinically relevant, as a meta-analysis by Deb et al. demonstrated that while D1 and D2 biopsies have comparable diagnostic yields (77.4% and 75.3%, respectively), inclusion of D1 resulted in a 6.9% increase in diagnostic yield, highlighting the importance of sampling from multiple duodenal sites to optimize diagnostic accuracy. ^22^

Our study found significant associations between CD and endoscopic and histological features such as villous atrophy, mucosal flattening, scalloping of duodenal folds, and mild increases in IEL counts ( *p* = 0.001). Villous atrophy and increased IELs are established indicators of CD, while elevated IgA anti-TG2 antibodies remain the gold standard for diagnosis. ^11^ Studies indicate that the diagnostic utility of IgA anti-TG2 antibodies can vary, with reduced sensitivity in certain populations or in those with selective IgA deficiency. ^23^ Other serological factors, such as EMA and DGP, showed no significant associations, likely due to their lower sensitivity in early-stage CD. ^24^

The lack of significant association between clinical symptoms and histological findings in our study aligns with recent research, such as Galli et al., which found no correlation between persistent GI symptoms and histological severity or serological markers, suggesting that symptom resolution may not always reflect histological healing. ^25^ Our study also revealed a significant association between mucosal flattening and gender, with males showing a higher prevalence. This aligns with previous research indicating that females have a 3.39 times higher likelihood of a longer symptom duration and malabsorption signs before a CD diagnosis. ^26^

Our study did not find significant associations between other endoscopic and histological features and gender or age, contrasting with literature suggesting age-related differences in CD presentations. For instance, a study found more pronounced clinical and histological features in children at initial diagnosis. ^27^ Another study reported that classical CD presentations are independently linked to age and year of diagnosis, rather than specific symptoms or histological findings. ^28^ This contrasts with Al-Qahtani et al. (2023), who found a significant association between gender and histopathological grading and classification of CD lesions. ^29^ Our analysis found no significant associations between clinical symptoms (e.g., malabsorption, extra-intestinal manifestations, comorbidities) and endoscopic or histological features. This contrasts with studies linking symptoms like anemia, fatigue, abdominal pain, and weight loss to villous atrophy on histopathology. ^30^

Specific villous atrophy in D1, mucosal flattening, and mild increases in IELs were significantly associated with positive IgA anti-TG2 antibodies ( *p* = 0.001, *p* = 0.003, *p* = 0.001, respectively), suggesting a correlation between IgA anti-TG2 levels and duodenal damage in CD. A meta-analysis by Qureshi (2023) also linked anti-TTG antibody levels to histologic severity in adolescents and adults. ^31^ Non-specific villous atrophy was significantly linked to positive anti-DGP antibodies, highlighting their diagnostic value in CD. A study found IgG anti-DGP to be a reliable test for CD in children, with high titers indicating severe duodenal atrophy. ^32^ High IgG anti-DGP titers correlated with severe duodenal atrophy, showing 95.4% sensitivity and 85.7% specificity for CD diagnosis. However, IgG anti-DGP without IgA anti-TG2 may indicate other GI conditions and has limited diagnostic value in children without clinical CD. ^33^

The strong association between positive IgA anti-TG2 antibodies and D1 villous atrophy, mucosal flattening, and mild IEL increase supports CD diagnosis. Husby et al. (2019) emphasized that a positive anti-TG2 warrants biopsy and histological analysis to confirm CD, as these antibodies indicate characteristic mucosal damage. ^23^ Our finding that all anti-DGP-positive participants had mild IEL increases met Marsh criteria, and showed villous atrophy and lymphocyte infiltration aligns with CD pathophysiology. In CD, gliadin peptide deamidation enhances immunogenicity, triggering immune responses that cause these histological changes. ^34^

Our study found GFD response rates of 7.1% overall and 20% for CD patients, lower than previously reported. A study of 382 biopsy-confirmed CD patients found 93.2% adhered strictly to GFD, but 46.9% continued to experience symptoms. ^35^ Research shows strict GFD adherence ranges from 42% to 80%, depending on the method and definition used. ^36^ Notably, 88.8% of participants and 69.2% of CD patients had missing documentation on their GFD response. Several factors, such as inadvertent gluten exposure, adherence duration, and individual patient factors, may explain the discrepancy. For example, 18.4% of patients with strict GFD adherence tested positive for elevated anti-TTG antibodies, suggesting unintentional gluten consumption. ^37^ Additionally, psychological factors, such as stress from dietary restrictions and social challenges, can affect adherence and symptom manifestation. ^36^

This study’s strengths include its comprehensive design, combining clinical, serological, and histopathological data from a diverse cohort. The inclusion of both D1 and D2 biopsies ensures a thorough evaluation of diagnostic yield in suspected CD cases. A limitation is its retrospective nature, which may introduce selection bias and reliance on incomplete or inconsistent hospital records. Additionally, the study focuses only on patients with suspected CD who underwent biopsy, potentially excluding undiagnosed or asymptomatic cases. The use of a single hospital setting may also limit generalizability to other regions with different diagnostic practices.

## Conclusion

This study highlights the importance of comprehensive diagnostic approaches, clinical, serological, and histopathological data, in improving CD diagnosis. It emphasizes the value of duodenal biopsies, particularly in early or atypical cases. While IgA anti-TG2 and EMA remain crucial, histopathological features like villous atrophy and IEL count are closely linked to CD diagnosis. Future research should assess the effectiveness of duodenal biopsies in varied populations and regions with differing CD prevalence, as well as the impact of genetic factors and long-term gluten-free diet adherence on CD outcomes.

## Data Availability

The data underlying this article cannot be shared publicly due to patient confidentiality and institutional privacy regulations at King Fahad University Hospital. De-identified data may be made available from the corresponding author upon reasonable request and subject to Institutional Review Board approval. No publicly archived datasets were generated or analyzed for this study.
