# Supplementary material for: Histopathological Correlation in Suspected Celiac Disease: Linking Clinical, Serological, and Endoscopic Findings
Source: F1000Res. 2025 Dec 8;14:1372. [Version 1] doi: 10.12688/f1000research.173342.1 (PMC12865290; doi:10.12688/f1000research.173342.1)
Supplement: Peer review history

# Reviewer report: Reviewer response for version 1

**DOI:** 10.5256/f1000research.191149.r449976

**Published:** 2026-02-02

**Authors:** Alessandro Granito

**Affiliations:** 1 Department of Medical and Surgical Sciences, University of Bologna, Bologna, Italy

In this retrospective study, the authors aimed to evaluate the diagnostic contribution of biopsies from the duodenal bulb (D1) and distal duodenum (D2) for suspected celiac disease. Overall, data from 224 adult and pediatric patients with suspected celiac disease (CD) (mean age was 40.9 ± 16.5 years) were assessed, and clinical, serological [IgA anti-tissue transglutaminase (anti-TG2), anti-endomysial antibodies (EMA), deamidated gliadin peptide], and histopathological data were analyzed. From the clinical point of view, Malabsorption symptoms were present in 64.3%, and 29% had extra-intestinal manifestations. Comorbidities (e.g., type 1 diabetes, thyroid disorders, inflammatory bowel diseases (IBD), psoriasis) were reported in 16.1%, while 72.3% had none. Sixty-five patients (29%) were diagnosed with CD. Villous atrophy, mucosal flattening, and increased intraepithelial lymphocytes were significantly associated with CD. Positive anti-TG2 and EMA antibodies were found to correlate with Marsh grade. The authors concluded that their results highlight the value of integrating histology by combining D1 and D2 sampling and serology in evaluating classical and non-classical CD presentations.

 The study is of interest and addresses a topic of clinical relevance. However, some issues should be addressed. 

 -In my opinion, other than villous atrophy, a very crucial point in the histological assessment for CD diagnosis is the IEL count. The authors should report whether they have useda CD3+ specific staining immunohistochemistry assessment. The recommended IEL assessment for CD diagnosis should refer to CD3+ IEL.

 -Another crucial point is the proper intestinal biosy orientation to avoid misdiagnosis and villous atrophy artefact (false-positive).

 -From the clinical viewpoint, the authors should expand in the discussion the potential correlation between the severity of mucosal damage and the clinical/immunological features. Previous literature data have demonstrated that more severe mucosal damage is associated with increased mucosal permeability, causing an immune response against multiple antigens, leading to the production of multiple serum antibodies against microbial antigens, such as anti-Anti-Saccharomyces cerevisiae antibodies (ASCA), as previously demonstrated (Refer reference no.1). as well as autoantigens as anti-neuronal antibodies that have been found in CD patients with neurological disorders, as reported (Refer reference no.2). These important clinical/immunological correlations of serum antibodies with intestinal damage and extraintestinal manifestations, as demonstrated in previous studies, should be recalled improving the clinical impact of the study findings.

Is the work clearly and accurately presented and does it cite the current literature?

Partly

If applicable, is the statistical analysis and its interpretation appropriate?

Yes

Are all the source data underlying the results available to ensure full reproducibility?

Yes

Is the study design appropriate and is the work technically sound?

Partly

Are the conclusions drawn adequately supported by the results?

Partly

Are sufficient details of methods and analysis provided to allow replication by others?

Yes

Reviewer Expertise:

Clinical Immunology, autoimmunity

I confirm that I have read this submission and believe that I have an appropriate level of expertise to confirm that it is of an acceptable scientific standard, however I have significant reservations, as outlined above.

## References

[1] : Sera of Patients With Celiac Disease and Neurologic Disorders Evoke a Mitochondrial-Dependent Apoptosis In Vitro. *Gastroenterology* .2007;133(1) : 10.1053/j.gastro.2007.04.070 195-206 10.1053/j.gastro.2007.04.070 17631142

[2] : Anti‐Saccharomyces cerevisiae and perinuclear anti‐neutrophil cytoplasmic antibodies in coeliac disease before and after gluten‐free diet. *Alimentary Pharmacology & Therapeutics* .2005;21(7) : 10.1111/j.1365-2036.2005.02417.x 881-887 10.1111/j.1365-2036.2005.02417.x 15801923

# Reviewer report: Reviewer response for version 1

**DOI:** 10.5256/f1000research.191149.r445836

**Published:** 2026-01-20

**Authors:** Reshma G Kini

**Affiliations:** 1 Father Muller Medical College, Mangaluru, Karnataka, India

The research hypothesis is adequately stated and is relevant to the context. The methodology is appropriate for hypothesis. The results are clinically relevant and useful to the reporting pathologist. the special significance is the detailed histopathological analysis. Also highlighted is the multimodal approach to diagnosis which is of significance. This adds to the existing knowledge.

Is the work clearly and accurately presented and does it cite the current literature?

Yes

If applicable, is the statistical analysis and its interpretation appropriate?

Yes

Are all the source data underlying the results available to ensure full reproducibility?

Yes

Is the study design appropriate and is the work technically sound?

Yes

Are the conclusions drawn adequately supported by the results?

Yes

Are sufficient details of methods and analysis provided to allow replication by others?

Yes

Reviewer Expertise:

Dermatopathology, Medical Education

I confirm that I have read this submission and believe that I have an appropriate level of expertise to confirm that it is of an acceptable scientific standard.

# Reviewer report: Reviewer response for version 1

**DOI:** 10.5256/f1000research.191149.r445837

**Published:** 2026-01-14

**Authors:** Pragya Gautam Ghimire

**Affiliations:** 1 Nepalgunj Medical College and Teaching Hospital, Banke, Nepal

**Reviewer Report**

 This manuscript addresses an important clinical topic; however several major methodological limitations need to be addressed by author

**Major Comments**

 The sample size (n = 224) is lower than the calculated required sample size (n = 385). This limitation should be acknowledged, with discussion of its impact on statistical power and interpretation.

 Biopsy location is unspecified in 81.5% of cases, despite being central to the study objective. This substantially limits meaningful comparison between duodenal bulb (D1) and distal duodenum (D2) biopsies. The authors should consider reframing the objectives, attempting retrospective site clarification, or presenting sensitivity analyses excluding unspecified cases.

 Please consider the diagnostic criteria for celiac disease as they are not well defined in the manuscript. The manuscript should state how CD was diagnosed and explain the discrepancy between the 29% CD diagnosis rate and the observed low anti-TG2 positivity (4.9%).

 Follow-up data on gluten-free diet response are missing for 88.8% of participants. It should be clarified whether this reflects inadequate follow-up or poor documentation, and the implications for diagnostic confirmation should be discussed.

 Minor Comments

 The observed male predominance contrasts with existing literature and should be discussed.

 The reported 20% gluten-free diet response rate among CD patients is unusually low and warrants clarification.

 The exclusion criterion “pre-existing gastrointestinal conditions” should be clearly defined. What are those?

 Overall Assessment

 The manuscript is well written however requires much changes to be considered as mentioned. A limitations section should be well included, and conclusions should be appropriately done.

Is the work clearly and accurately presented and does it cite the current literature?

Yes

If applicable, is the statistical analysis and its interpretation appropriate?

Partly

Are all the source data underlying the results available to ensure full reproducibility?

Partly

Is the study design appropriate and is the work technically sound?

Yes

Are the conclusions drawn adequately supported by the results?

Partly

Are sufficient details of methods and analysis provided to allow replication by others?

Partly

Reviewer Expertise:

Histopathology, Genetics, hematology, cytology, hemoglobinopathy, tropical disease.

I confirm that I have read this submission and believe that I have an appropriate level of expertise to confirm that it is of an acceptable scientific standard, however I have significant reservations, as outlined above.
